# Is there a Role for Radical Surgery in Advanced Gallbladder Carcinoma?

**DOI:** 10.1155/1997/69825

**Published:** 1997

**Authors:** M. Margaret Kemeny

**Affiliations:** North Shore University Hospital Department of Surgery Cornell University Medical College 300 Community Drive Manhasset New York 11030 USA

## Abstract

Forty-four patients with advanced gallbladder carcinoma (18 with stage pT_3_ and 26 with stage pT_4_ of the Union Internacional Contra la Cancrum classification) were aggressively managed by extended heptatic resection in 33 patients, bile duct resection in 28, pancreaticoduodenectomy in seven, gastrointestinal resection in eleven and portal vein resection and reconstruction in seven. Adjacent organ involvement was classified as follows: type I, hepatic involvement with or without gastrointestinal invasion (Ia, Ib); type II, bile duct involvement with or without gastrointestinal invasion (IIa, IIb); type III, hepatic and bile duct involvement with or without gastrointestinal invasion (IIIa, IIIb); type IV, gastrointestinal involvement without hepatic or bile duct invasion. Fourteen of 15 patients with type I tumours had a curative resection compared with seven of 26 with type III lesions (*P*< 0.0001). The surgical mortality rate was two of 15 patients with type I tumours, seven of 26 with type III tumours and nine of 44 for the whole group. The long-term survival rate after curative resection was four and two of 23 at 3 and 5 years respectively, significantly better than two and none of 21 at I and 2 years after non-curative resection (*P*<0.01). The survival rate after curative resection for patients with type I tumours was four and two of 14 at 3 and 5 years respectively, significantly better than for other types (*P*<0.05). This classification of advanced gallbladder carcinoma according to involvement of adjacent organs might be helpful in planning surgery for this condition; in particular, type I tumours should be treated by a radical surgical procedure to achieve a favourable outcome.


gastropathy by portacaval shunt. *Hepatology*, **21**, 1011–1017.
[2] Mcormack, T. T., Sims, J. and Eyre-Brook, I. *et al.* (1985). Gastric lesions in portal hypertension: Inflammatory gastritis or congestive gastropathy? *Gut*, **26**, 1226–1232.
[3] D'Amico, G., Montalbano, L. and Traina, M. *et al.* (1990). Natural history of congestive gastropathy in cirrhosis. *Gastroenterology*, **99**, 1558–1564.
[4] Sarfeh, I. J. and Tarnawski, A. (1992). Portal hypertensive gastropathy. *Problems in General Surgery*, **9**, 431–435.
[5] Sarfeh, I. J., Tabak, C., Eugene, J. and Juler, G. L. (1981). Clinical significance of erosive gastritis in patients with alcoholic liver disease and upper gastrointestinal hemorrhage. *Ann. Surg.*, **194**, 149–151.
[6] Sarfeh, I. J., Juler, G. L., Stemmer, E. A. and Mason, G. R. (1982). Results of surgical management of hemorrhagic gastritis in patients with gastroesophaageal varices. *Surg. Gynecol. Obstet.*, **155**, 167–170
[7] Babb, R. R. and Mitchell, R. L. (1988). Persistent hemorrhagic gastritis in a patient with portal hypertension and esophagogastric varices: The role of portal decompressive surgery. *Am. J. Gastroenterol.*, **83**, 777–779.
[8] Hashizumi, M., Tanaka, K. and Inokuchi, K. (1983). Morphology of gastric microcirculation in cirrhosis. *Hepatology*, **3**, 1008–1012.
[9] Tarnawski, A., Sarfeh, I. J., Bui, H. X. and Stachura, J. (1988). Microvascular abnormalities of the portal hypertensive gastric mucose. *Hepatology*, **8**, 1488–94.
[10] Ichikawa, Y., Tarnawski, A. and Sarfeh, I. J. *et al.* (1994). Distorted microangioarchitecture and inpaired angiogenesis in gastric mucose of portal hypertensive rats. *Gastroenterol*, **106**, 702–708.
[11] Sarfeh, I. J., Soliman, K. and Waxman, K. *et al.* (1989). Impaired oxygenation of gastric mucosa in portal hypertension: The basis for increased susceptibility to injur. *Dig. Dis. Sci.*, **43**, 225–228.
[12] Sarfeh, I. J. and Tarnawski, A. (1991). Increased susceptibility of the portal hypertensive gastric mucosa to damage. *J. Clin. Gastroenterol.*, **13**, (Suppl. 1): S18–S21.
[13] Spina, G. and Arcidiacono, R. [eds.] (1994). Gastric Endoscopic Features in Portal Hypertension: Proceedings of the Consensus Conference of the New Italian Endoscopic Club (NIEC), Milan, Milano: Masson S.p.A.
[14] Conn, H. O. (1995). Emergency portacaval anastomosis in portal hypertensive gastropathy: Another piece of the puzle. *Hepatology*, **21**, 1190–1192.

John Craig Collins, MD and
I James Sarfeh, MD
Veterans Affairs Hospital and
Department of Surgery
University of California,
Irvine 101 The City Drive
Orange, California 92668-9969
United States of America

### Is there a Role for Radical Surgery in Advanced Gallbladder Carcinoma?

### ABSTRACT

*Miyazaki, M., Itoh, H., Ambiru, S., Shimizu, H., Togawa, A., Gohchi, E., Nakajima, N. and Suwa, T. (1996) Radical surgery for advanced gallbladder carcinoma. British Journal of Surgery; 83, 478 – 481.*

**Forty-four patients with advanced gallbladder carcinoma (18 with stage $pT_3$ and 26 with stage $pT_4$ of the Union Internacional Contra la Cancrum classification) were aggressively managed by extended heptatic resection in 33 patients, bile duct resection in 28, pancreaticoduodenectomy in seven, gastrointestinal resection in eleven and portal vein resection and reconstruction in seven. Adjacent organ involvement was classified as follows: type I, hepatic involvement with or without gastrointestinal invasion (Ia, Ib); type II, bile duct involvement with or without gastrointestinal invasion (IIa, IIb); type III, hepatic and bile duct involvement with or without gastrointestinal invasion (IIIa, IIIb); type IV, gastrointestinal involvement without hepatic or bile duct invasion. Fourteen of 15 patients with type I tumours had a curative resection compared with seven of 26 with type III lesions ($P< 0.0001$). The surgical mortality rate was two of 15 patients with type I tumours, seven of 26 with type III tumours and nine of 44 for the whole group. The long-term survival rate after curative resection was four and two of 23 at 3 and 5 years respectively, significantly better than two and none of 21 at 1 and 2 years after**

**non-curative resection ($P<0.01$). The survival rate after curative resection for patients with type I tumours was four and two of 14 at 3 and 5 years respectively, significantly better than for other types ($P<0.05$). This classification of advanced gallbladder carcinoma according to involvement of adjacent organs might be helpful in planning surgery for this condition; in particular, type I tumours should be treated by a radical surgical procedure to achieve a favourable outcome.**

*Keywords:* Pancreaticogastrostomy, pancreaticojejunostomy, pancreaticoduodenectomy

### PAPER DISCUSSION

Surgery for advanced gallbladder cancer remains a controversial topic because of the poor prognosis and lack of controlled studies. "Radical surgery for advanced gallbladder cancer" presents the authors' surgical experience with 44 patients who had advanced gallbladder cancer. All 44 patients were at least a Nevin's Stage III or IV cancer. They were further subdivided by the authors into four different types: 1. hepatic type, 2. biliary type, 3. hepatobiliary type and 4. others. By using this subdivision, the operative mortality of the procedures could be analyzed in a more meaningful fashion. Of the 15 patients with Type 1 tumors, two (13%) had a surgical mortality and 14 (93%) had an attempt at curative resection. In contrast, only 7 (27%) of the 26 patients with Type III lesions had a curative resection with 7 (27%) having a surgical mortality. The only two patients of the whole series of 44 patients that survived for more than five years were in the Type 1 group.

The operative mortality for patients in both groups was poor. Overall for the whole group, the surgical mortality was 20%, and only 23 of the 44 patients had attempted curative resections (52%).

The long term survival rate, after curative resection which was done in only 23 patients (52%), was four patients at three years, and two patients at five years, an 8.7% overall five year survival. This report underscores the dismal prognosis with gallbladder cancer where only two out of 44 patients survived for five years. Furthermore, it is not even noted if those two patients are disease free. The authors are trying to make a case for the fact that the two patients who did survive had Type 1 tumors. This is a 14% five year survival for this type, but it still remains dismal. More importantly, it is upsetting to note that even for the Type 1 tumors, the survival and the mortality from the operation are almost exactly equal numbers. For the Type 2 group, there is no five year survival and the mortality is 26%.

Although the authors are trying to select the patients who should have radical surgery, I think that this paper points out that radical surgery should not be done at all. When radical surgery has a 30 day mortality that is greater than the possible survival benefit, its usefulness must be questioned. Especially in this period of renewed efforts with new Phase 1 chemotherapy agents, it might be much more efficacious for a patient to have a chance at a response with chemotherapy rather than die from an operation.

Furthermore, this study did not describe the prognostic benefit of the lymph nodes status. Since lymph node involvement is another sign of advanced tumor, it might be interesting to know if the patients who had longer survival had positive lymph nodes or not. This is not mentioned in the text of the paper.

The literature on gallbladder surgery is moderately sparse because of the lack of large studies on this disease and because of the very poor survival after surgery for this disease. One of the largest U.S. studies of gallbladder cancer is a review from the Mayo Clinic of all the patients treated surgically at that institution for a 12 year period [1]. Of the 111 patients who had surgery, 20% had a biopsy only and another 27% had a palliative cholecystectomy and 20 patients had radical resections with no mortality. Although overall, there was no difference in survival for the patients having the cholecystectomy over the radical resection; this was not a randomized series and the patients in the cholecystectomy group were those with the more favorable disease stage. In the radical surgery group 3 patients with Nevin's Stage 3, and two with Nevin's Stage 4 survived, and the only patient in the group with Nevin's Stage 2 survived. The conclusions we can make from these kinds of studies are limited. Some patients will benefit from radical resection, but it is not clear which ones. Mortality from radical surgery must, however, be low enough to equal the possible benefit.

Currently it is believed that patients with Nevin's Stage 1 and in situ disease, that is a disease that is limited to the mucosa, have a 60% chance of survival [2]. Whether those patients would do better with more radical operations remains controversial. Patients with Nevin's Stage 2 disease only have a survival of around 20% [3]. One must keep in mind that Stage 2 disease is a disease that is still in the wall of the gallbladder. Here again, the controversy over whether doing a more radical cholecystectomy which includes removal of the liver bed and dissection of the periportal lymph nodes is unclear and no randomized prospective study has been done.

The present paper points out that in patients with disease that is beyond Stage 2, which is the case for all of these patients, a chance of cure is very minimal and surgery should be embarked upon only in very selected cases. Perhaps looking at the criteria that these authors have delineated may offer some guidelines for the future. Patients with the Type 3 disease delineated by the authors, that is disease involving both the liver and the extrahepatic bile ducts with or without intestinal involvement, do not do well with surgery. They have unacceptable mortality in the authors series of over 26%. In the figure that shows the survival curves for the seven patients with Type III tumors that had curative resections, the mean survival seems to be less than six months, which is not that different than the 124 day (four months) mean, survival for patients with non-curative resections. These patients should not be offered surgical resection since their mortality far exceeds the possible curative surival benefits.

Whether or not patients with the Type 1 disease, i.e. disease which extends into the liver or without intestinal involvement, should have surgery is a very difficult question to answer [4,5]. It seems at first glance that with a mortality of 8.7% and a five year survival of 14%, the benefit is negligible. However, there may be a survival benefit for this group overall, not a five year survival benefit, but a mean survival benefit. Unfortunately, this cannot be deduced from this report since the only information on this is in Figure 3 which is inaccurate since it shows the Type 1 patients having a 40% five year survival, which we know is different than the text.

In conclusion, the case for surgery in patients with gallbladder cancer very much remains an open issue. For patients with earlier cancer, the question still is how radical should the surgery be. For patients with advanced disease, the usefulness of surgery seems minimal at best. All of the information underscores the need for better treatments for this type of cancer and a more multidisciplinary approach. Also, a multinational protocol to investigate treatments for gallbladder cancer could be very important for any improvements in the future.

### *References*

[1] Donohue, J. H., Nagorney, D. M. and Grant, C. S. *et al.* (1990). Carcinoma of the gallbladder, *Arch. Surg.*, **125**, 237–241.

[2] White, K., Kraybill, W. G. and Lopez, J. (1988). Primary cacinoma of the gallbladder: TNM staging and prognosis, *J. Surg. Onc.*, **38**, 25–255.

[3] Abelo, M. D., Armitage, J. O., Lichter, A. S. and Niederhuber, J. E. (eds) (1995). *Clinical Oncology*. Churchill Livingston Inc.
[4] Ouchi, K., Suzuki, M. and Saijo, S. *et al.* (1993). Do recent advances in diagnosis and operative management improve the outcome of gallbladder? *Surgery*, **113**(3), 324–329.
[5] Wanebo, H. J., Castle, W. N. and Fechner, R. E. (1982). Is carcinoma of the gallbladder a curable lesion? *Ann. Surg.*, **195**, 624–631.

M Margaret Kemeny, MD
North Shore University Hospital
Department of Surgery
Cornell University Medical College
300 Community Drive
Manhasset
New York 11030
United States of America

### Mini-Incision Versus Laparoscopic Cholecystectomy

### ABSTRACT

*Majeed, A. W., Troy, G., Nicholl, J. P., Smythe, A., Reed, M. W. R., Stoddard, C. J., Peacock, J. and Johnson A. G. (1996) Randomised, prospective, single-blind comparison of laparoscopic versus small-incision cholecystectomy. The Lancet; 347*, 989–994.

***Background:*** **We report a prospective randomised comparison between laparoscopic and small-incision cholecystectomy in 200 patients which was designed to eliminate bias for or against either technique.**
***Methods:*** **Patients were randomised in the operating theatre and anaesthetic technique and pain-control methods were standardised. Four experienced surgeons did both types of procedure. Identical wound dressings were applied in both groups so that carers could be kept blind to the type of operation.**
***Findings:*** **There was no significant difference between the groups for age, sex, body mass index, and American Society of Anaesthesiologists grade. Laparoscopic cholecystectomy took significantly longer than small-incision cholecystectomy (median 65 [range 27–140] min *vs* 40 [18–142] min, $p<0.001$). The operating time included operative cholangiography which was attempted in all patients. We found no significant difference between the groups for hospital stay (postoperative nights in hospital, median 3.0 [1–17] nights for laparoscopic *vs* 3.0 [1–14] nights for small-incision, $p=0.74$), time back to work for employed persons (median 5.0 weeks *vs* 4.0 weeks; $p=0.39$), and time to full activity (median 3.0 weeks *vs* 3.0 weeks; $p=0.15$).**
***Interpretation:*** **Laparoscopic cholecystectomy takes longer to do than small-incision cholecystectomy and does not have any significant advantages in terms of hospital stay or postoperative recovery.**

*Keywords:* Laparoscopic cholecystectomy, mini-incision cholecystectomy

### PAPER DISCUSSION

Laparoscopic cholecystectomy serves as the prototype success story for the introduction of minimally invasive surgery into the mainstream practice of surgery world wide. This study by Majeed *et al.* recruited 200 patients over a three and one-half year period with symptomatic gallstones. Patients were randomized intra-operatively after the induction of anesthesia to undergo either standard laparoscopic cholecystectomy with an attempt at routine operative cholangiography, or "small-incision" cholecystectomy. The small incision used in this study was a high transverse sub-xiphoid incision, dividing the rectus muscle as needed, and dissecting the gallbladder from Calot's triangle toward the fundus with long instruments, avoiding the insertion of hands into the peritoneal cavity. An important aspect of this study is that the patients were treated post-operatively with a patient-controlled analgesic system delivering morphine, and that the patients were "told that they could get out of bed and go home as soon as they felt fit

